# Clinical utility of the UCSD Performance-Based Skills Assessment—Brief (UPSA-B) in adults living with HIV: Associations with neuropsychological impairment and patient-reported everyday functioning difficulties

**DOI:** 10.1371/journal.pone.0183614

**Published:** 2017-08-24

**Authors:** Raeanne C. Moore, Emily W. Paolillo, Anne Heaton, Pariya L. Fazeli, Dilip V. Jeste, David J. Moore

**Affiliations:** 1 Department of Psychiatry, University of California San Diego, San Diego, California, United States of America; 2 VA San Diego Healthcare System, San Diego, California, United States of America; 3 San Diego State University/University of California San Diego, San Diego Joint Doctoral Program in Clinical Psychology, San Diego, California, United States of America; 4 UAB School of Nursing, The University of Alabama at Birmingham, Birmingham, Alabama, United States of America; 5 Stein Institute for Research on Aging, University of California San Diego, San Diego, California, United States of America; The University of New South Wales, Neuroscience Research Australia, AUSTRALIA

## Abstract

**Objective:**

Requiring only 10–15 minutes to complete, the UCSD Performance-Based Skills Assessment (UPSA-B) has high clinical utility as a brief measure of functional capacity. This study aimed to validate the UPSA-B in adults living with HIV/AIDS (HIV+), and identify whether the UPSA-B can be used as an indicator of functional dependence in this population.

**Method:**

One hundred and three HIV+ adults and 91 HIV- adults completed a comprehensive neuropsychological and neuromedical battery, including a self-report measure of functional status (IADL Dependence vs. IADL Independence), an objective measure of functional capacity (UPSA-B), and a self-report measure of mood states including a subscale related to cognitive difficulties (Profile of Mood States [POMS]-Confusion/Bewilderment subscale).

**Results:**

HIV+ participants had significantly lower UPSA-B scores than their HIV- counterparts (*p* = 0.02), although this fell to a trend (*p* = 0.08) when including covariates. Among the HIV+ group, higher UPSA-B scores were related to better neuropsychological ability, but unrelated to self-reported functional independence. Conversely, UPSA-B scores were unrelated to participant-reported cognitive difficulties on the POMS Confusion/Bewilderment subscale. An ROC curve was generated to determine the optimal UPSA-B value for discriminating between normal neuropsychological functioning versus neuropsychological impairment, with results indicating an optimal cutoff of 79. The UPSA-B identified HIV+ persons with cognitive impairment with 70.9% accuracy.

**Conclusions:**

The UPSA-B was able to differentiate neuropsychological impairment from no impairment among HIV+ participants and holds promise as a clinical screening tool in this population. However, indicators of functional disability among adults living with HIV is still not well understood and is likely multifactorial in nature. These data highlight the complex interplay between objective functional capacity, neurocognitive ability, subjective cognitive symptoms, and functional dependence.

## Introduction

HIV is a chronic illness frequently resulting in diminished capacity to function independently [1–3]. Such impairments in everyday (“real-world”) functioning are often characterized by declines in basic and instrumental activities of daily living (e.g., housekeeping, cooking) [1], impaired driving ability [4], poor HIV disease management [2,5], increased difficulties in vocational functioning [1,6], and increased rates of unemployment [1,2,7,8]. In some regions, including the United States and South Africa, these HIV-related functional impairments contribute to severe economic burden, as decreases in productivity and labor supply may significantly decrease gross domestic product growth rate [9,10]. Additionally, the reduced ability to care for oneself and properly manage HIV disease (e.g., medication management/adherence) not only poses an individual risk for accelerated disease progression and mortality [11], but also poses a notable public health concern (e.g., the possibility of higher HIV transmission).

Everyday functioning impairments among people living with HIV/AIDS may be due to one or a combination of several factors. Presence of HIV-associated neurocognitive disorders (HAND) are one of the strongest and most consistent correlates of everyday functioning difficulties in the context of HIV/AIDS, with neuropsychologically impaired individuals often performing worse on domains of functional ability compared to neuropsychologically intact individuals [1,3,6,12,13]. Specifically, impairment on neuropsychological (NP) ability domains of executive function, learning, attention/working memory, and verbal abilities are most strongly associated with functional impairment [1]. Functional ability among individuals living with HIV/AIDS is also related to other factors such as mood (e.g., depression) [1,14], substance use [7], and inadequate motivation (e.g., apathy) [15]. This differs from other clinical populations in which functional impairments are more strongly and directly tied to cognitive deficits (e.g., Alzheimer’s disease) [16]. As such, there may be several types of assessments by which we can identify those at risk for functional impairments in the HIV/AIDS clinical population.

Healthcare providers are in need of accurate methods to determine whether someone has basic skills needed to live independently or participate in vocational activities. Currently, researchers and clinicians use several different methods for measuring everyday functioning in people living with HIV/AIDS. One widely accepted and used measure for determining if an individual has the abilities necessary for independent living (i.e., functional independence) is the self-reported Lawton and Brody instrumental activities of daily living (IADL) questionnaire [1,17]. This measure assesses the level of competency with which individuals are able to perform everyday tasks (e.g., housekeeping, managing finances, buying groceries), if they have difficulty, or need assistance to do these tasks. When using the IADL questionnaire, functional dependence is determined when individuals report substantial difficulties or needing substantial assistance to successfully perform these critical everyday activities. Additionally, self-reported recent feelings of difficulties with cognition (e.g., Profile of Mood States [POMS] Confusion/Bewilderment subscale) [18] are commonly used to determine perceived cognitive difficulties, and have shown to be associated with functional abilities [14]. While these self-report measures are prone to retrospective recall and other errors due to cognitive deficits, state-dependent bias (e.g., depressed mood), or level of insight, they are easy to administer and brief, making them commonly used and appropriate for clinical settings. Objective NP functioning may also be used to predict functional impairment [1]; however, a comprehensive NP assessment is time consuming and may not always be feasible as a measure of functional ability. Moreover, cognitive impairment is not always indicative of functional impairment among persons living with HIV [19]. On the other hand, objective performance-based measures of functional capacity that are brief and easy to administer may hold the most promise as relatively quick, ecologically valid, and accurate direct measures of functional impairment.

One such objective measure of functional capacity is the Brief UCSD Performance-Based Skills Assessment (UPSA-B). This measure was originally developed for assessment of everyday functioning in adults with severe mental illness [20,21], and has been validated as an adequate screener and predictor of functional independence and employment in schizophrenia and bipolar disorder [22–25]. Since its development, the UPSA-B has become a widely used measure in populations of patients with severe mental illness, with several versions that have been regionally/culturally adapted and validated, such as the European Spanish version [26], Swedish version [27], and Japanese version [28]. Computerized and mobile versions of the UPSA-B (i.e., the C-UPSA and UPSA-M, respectively) have also been developed and validated for use in schizophrenia [29,30]. Beyond the severely mentally ill population, however, research on the UPSA in other patient populations is scant [31,32]. Furthermore, the UPSA-B has yet to be examined as a valid measure of functional impairment in people living with HIV/AIDS, although subcomponents of the UPSA have been incorporated into the Multitasking Test, a test which has been shown to be predictive of IADL dependence among HIV+ adults [2].

The current study aims to examine the associations between UPSA-B performance, objective NP functioning, IADL dependence, and self-reported cognitive difficulties among persons living with HIV in order to determine whether the UPSA-B can be used as a measure of everyday functioning outcomes among persons living with HIV, which has not previously been examined in the literature. We hypothesized that HIV+ adults would perform significantly worse on the UPSA-B compared to HIV- adults. To test the convergent and discriminant validity of the UPSA-B among people living with HIV/AIDS, we examined the relationships between UPSA-B performance with global NP ability, IADL dependence, and self-reported cognitive difficulties, with the hypothesis that UPSA-B performance would be an adequate indicator of NP impairment and IADL dependence. Lastly, based on literature indicating that self-reported cognitive difficulties are generally unrelated to objective cognitive impairment [14,19,33] and UPSA-B performance is related to objective cognitive impairment, we hypothesized cognitive difficulties would be unrelated to UPSA-B scores. If UPSA-B performance is a stronger indicator of IADL dependence than lengthy comprehensive NP testing or self-reported cognitive difficulties, the results of the current study may provide support for implementation of brief measure(s) to better characterize and assess for HIV-associated functional impairments in both research and clinical settings where such functional determinations are critical for identification of individuals in need of further treatment, care, and clinical/functional support.

## Methods

### Participants and design

Participants included 103 HIV+ and 91 HIV- adults from the Multi-Dimensional Successful Aging among HIV-Infected Adults study conducted at the University of California, San Diego (UCSD). Baseline data were examined in this study. Participants were matched for age and race/ethnicity, but not sex or education. The UCSD Institutional Review Board approved this study, and all participants provided written, informed consent. Exclusion criteria for HIV+ and HIV- individuals were minimal: 1) diagnosis of a psychotic disorder or mood disorder with psychotic features; 2) presence of a neurological condition (other than HIV infection) that could negatively impact cognitive functioning (e.g., Alzheimer’s disease, stroke, traumatic brain injury); and 3) positive urine toxicology for alcohol or drugs (excluding marijuana) on the day of testing. All participants were tested for HIV infection using an HIV/HCV finger stick point of care test (Abbott RealTi*m*e HIV-1 test, Abbott Laboratories, Illinois, USA). No participants who reported being HIV- at screening tested positive for HIV or Hepatitis C (HCV).

Participants were reviewed for severe confounding neuromedical conditions, not captured by the exclusion criteria or screening, that might negatively affect neurocognitive and everyday functioning and hinder our ability to attribute impairment to direct effects of HIV. We started with 116 HIV+ participants, but 13 participants were classified as severely confounded (e.g., cerebrovascular accident, myocardial infarction, congestive heart failure, and/or moderate-to-severe liver disease) and excluded from analyses, which gave us the final HIV+ sample size of 101 (Mean age = 50.3 years; SD = 8.5; range: 35–65 years) used in the present study. One of the HIV- participants had a severe confound yielding a sample size of 91 HIV- for the present study.

### Measures

#### Neuropsychological battery

A standard HIV Neurobehavioral Research Program (HNRP) neuropsychological battery covering seven neurocognitive domains was used in the present study [1]. This battery was designed in accordance with international consensus conference recommendations and has published norms that correct for demographic effects [age, education, sex, and (when appropriate) race/ethnicity] [34,35]. Cognitive domains assessed include: verbal fluency, attention/working memory, abstraction/executive functioning, learning, memory (delayed recall), speed of information processing, and complex motor skills [35]. Table 1 contains the individual neuropsychological tests that comprise this battery. Raw scores from the neuropsychological tests were converted to demographically-adjusted T-scores (M = 50, SD = 10 in healthy subjects). Global and domain-specific continuous T-scores were used in our comparisons of cognition with other study variables.

**Table 1:** Tests in the neuropsychological battery.

Cognitive Domain	Tests
Speed of Information Processing	WAIS-III Digit Symbol WAIS-III Symbol Search Trail Making Test, Part A Stroop Color Trial
Learning and Memory (2 domains)	Hopkins Verbal Learning Test-Revised Brief Visuospatial Memory Test-Revised
Abstraction/Executive Functioning	Wisconsin Card Sorting Test (64-item) Trail Making Test, Part B Stroop Color Word Trial
Verbal Fluency	Controlled Oral Word Association Test Category Fluency (Animals) Category Fluency (Actions)
Attention/Working Memory	WAIS-III Letter-Number Sequencing PASAT (1^st^ channel only)
Motor	Grooved Pegboard Test (Dominant & Non-dominant Hands)

*Note*. WAIS-III: Wechshler Adult Intelligence Scale 3^rd^ Edition; PASAT: Paced Auditory Serial Addition Task.

To derive domain-based and global deficit scores (GDS), T-scores were subsequently converted into averaged deficit scores, ranging from 0 (no impairment) to 5 (severe impairment) [36]. A GDS Impairment variable is created such that participants with GDS scores greater than 0.5 are deemed neurocognitively impaired, whereas GDS scores less than or equal to 0.5 are deemed to have cognition within normal limits. The GDS Impairment variable was used in the ROC analysis. Lastly, premorbid intellectual functioning was assessed via the Wide Range Achievement Test 4^th^ Edition (WRAT-4) Word Reading subtest.

#### Everyday functioning measures

Functional Dependence: Functional dependence vs. independence in instrumental activities of daily living (IADLs) was determined using a modified version of the Lawton and Brody scale (Heaton et al., 2004). The IADL questionnaire includes questions of managing finances, using the telephone, cooking, buying groceries, working, transportation, understanding of written and viewed material, social activities, and childcare. The IADL is a self-report measure that asks participants to answer questions relating to the level of assistance needed currently compared to when they were at their highest level of functioning. If a decline in functioning in at least two domains was reported, participants were classified as IADL Dependent. This IADL classification approach has been validated in a normative sample compared to one with cognitive impairment [1]. Individuals classified as both NP impaired and IADL Dependent would be diagnosed as having Mild Neurocognitive disorder (MND) or HIV-associated Dementia (HAD) depending on severity of neuropsychological impairments, whereas NP impaired participants without IADL dependence would be diagnosed as having Asymptomatic Neurocognitive Impairment (ANI; i.e. Frascati criteria)[34].

Performance-Based Measure of Functional Capacity: The UCSD Performance-Based Skills Assessment-Brief (UPSA-B) [21,24] was used to assess participants’ capacity to perform tasks similar to those encountered in daily life. The UPSA-B was developed through the use of factor analysis (20) and consists of two of the five subscales from the full UPSA: 1) *Financial skills* and 2) *Communication skills*. As opposed to the Computerized UPSA (C-UPSA; 29) and UPSA Mobile App (UPSA-M; 30), the UPSA-B uses tangible props to simulate real-world tasks. This task requires approximately 10–15 minutes to complete. Assessment of financial skills involves tasks in which participants handle fake money (i.e., dollar bills, coins, and checks) to count, make change, and pay bills. Assessment of communication skills involves tasks in which participants use a disconnected landline telephone to simulate making calls (e.g., directory assistance, doctor’s office) to communicate requested or necessary information. In a review of instruments for measuring functional recovery in those diagnosed with serious mental illness, the UPSA-B received the highest overall rating (25). Total percent correct is calculated for each subscale and converted to a standardized score ranging from 0–50. A summary score is then calculated by summing the two domain scores for a total score ranging from 0–100, with higher scores indicating better functional capacity.

#### Participant-reported cognitive difficulties

The Profile of Mood States (POMS) questionnaire assesses psychological distress. This measure consists of six subscales: Tension-Anxiety, Depression-Dejection, Anger-Hostility, Vigor-Activation, Fatigue-Inertia, and Confusion-Bewilderment [18]. The 7-item Confusion-Bewilderment subscale, which includes items on forgetfulness, problems concentrating, and muddled or uncertain thoughts, was used in this study to assess subjective cognitive functioning (i.e., cognitive difficulties). Participants rate their experience of cognitive difficulties during the past week on a scale from 0 = *not at all* to 4 = *extremely*, for a total subscale score ranging from 0–28. Higher scores indicate more symptoms.

#### HIV disease characteristics

All participants completed a standardized research neuromedical evaluation, including a thorough medical history, assessment of current HIV symptoms and antiviral medications, a physical and neurological evaluation, CDC staging, and a blood draw. For individuals with HIV-infection, HIV RNA viral load (VL) was measured by reverse transcriptase-polymerase chair reaction (Abbott m2000 HIV 1,2; lower limit of quantitation 40 copies per milliliter).

*Statistical Analyses*. Data were examined for normality and three sets of analyses were conducted. First, *t*-tests were performed to determine group differences on the UPSA-B total scores and each of the two domains of functioning measured on the UPSA-B (finances; communication). Next, among the HIV+ group, the following analyses were performed (1): Pearson correlations were performed to determine the relationships between global neuropsychological ability (i.e., global T-scores), UPSA-B scores, and POMS Confusion/Bewilderment scores, and 2) Additional *t*-tests were performed to determine if UPSA-B and POMS scores differed based on the presence or absence of functional dependence.

Based on results of the first analyses, we plotted a receiver operating characteristic (ROC) curve for the UPSA-B to determine the optimal cutoff in discriminating between cognitive impairment vs. no cognitive impairment (using the GDS Impairment variable). The optimal cutoff score was determined by producing a Yuden’s index value, which is one minus specificity subtracted from sensitivity [37,38]. The area under the curve (AUC) with 95% confidence intervals (CI) was used as an indicator of the ability of the UPSA-B to differentiate between neurocognitive status.

## Results

### Comparison of HIV+ and HIV- performance on the UPSA-B

See Table 2 for full descriptive statistics of our sample. HIV+ participants’ total score on the UPSA-B was significantly lower than the HIV- participants (*p* = 0.02). The HIV+ participants performed significantly worse in the Financial Skills subtest compared to the HIV- participants, and their performance was lower than the HIV- participants in Communication Skills subtest at a trend-level (see Table 3). The groups did not differ on age or race/ethnicity. Given that the HIV- group was significantly more educated and had more females than the HIV+ group, we repeated these analyses covarying for sex and education. When these demographic variables were controlled for, UPSA-B total scores did not significantly differ between the groups (F = 3.19, *p* = 0.08), although a trend-level finding was still observed. The HIV+ group had significantly lower global neuropsychological scores, had a greater rate of IADL dependence, and had more cognitive difficulties than the HIV- group (see Table 2).

**Table 2:** Demographic and clinical characteristics of the participants.

	HIV+ participants (n = 103)	HIV- participants (n = 91)	*t* or *Chi*^*2*^	*df*	*p*
**Demographic Characteristics**					
Age (yrs), M(SD)	50.3(8.5)	51.4(7.6)	0.95	192	0.35
Education (yrs), M(SD)	14.0(2.2)	15.0(2.3)	2.82	192	<0.01
*Gender*			4.84	1	0.03
Male, *n*(%)	87(84.5)	65(71.4)			
Female, *n*(%)	16(15.5)	26(28.6)			
*Race/Ethnicity*			1.70	1	0.19
White, *n*(%)	62(61.4)	64(70.3)			
Other, *n*(%)	39(38.6)	27(29.7)			
**HIV Disease Characteristics**					
Est. duration of HIV (yrs), M(SD)	17.0(8.8)	—	—	—	—
AIDS, *n*(%)	61(59.2)	—	—	—	—
cART^c^, *n*(%)	92(89.3)	—	—	—	—
Nadir CD4^a^ (cells/ μl)	181.5(58.0,353.0)	—	—	—	—
Current CD4^a^ (cells/ μl)	628.5(432.0,846.3)	—	—	—	—
Plasma detectable, *n*(%)^b^	5(6.1)	—	—	—	—
**Medical Characteristics**					
Hepatitis C, *n*(%)	21(20.4)	0(0)	20.81	1	<0.01
Body Mass Index, M(SD)	27.5 (6.0)	27.4 (6.6)	0.10	188	0.92
SF-36 Physical Health Composite Score, M(SD)	70.1(24.1)	84.2(18.9)	4.46	188	<0.01
**Psychiatric Characteristics**					
Current Major Depressive Disorder, *n*(%)	12(11.7)	0(0)	12.00	1	<0.01
Alcohol dependence^d^, *n*(%)	32(31.1)	9(10.1)	13.02	1	0.001
Non-alcohol dependence^d^^,^^e^, *n*(%)	47(46.5)	8(9.0)	32.44	176	<0.001
**Cognition and Daily Functioning**					
Premorbid IQ^f^, M(SD)	103.8(13.8)	106.9(13.6)	1.58	190	0.12
GDS impairment, *n*(%)	40(38.8)	23(25.3)	4.05	1	0.04
IADL dependence, *n*(%)	27(26.2)	5(5.5)	15.06	1	<0.001
POMS, M(SD)	7.0(5.7)	3.4(3.4)	5.24	190	<0.001

Note.

^a^Data represent medians with interquartile ranges in parentheses;

^b^n = 82;

^c^5 additional participants are a non-cART regimen;

^d^Denotes a lifetime diagnosis;

^e^includes cannabis, cocaine, hallucinogens, inhalants, methamphetamine, opioids, PCP, or other substances;

^f^measured with the Wide Range Achievement Test (WRAT); IADL = Instrumental Activities of Daily Living; POMS = Profile of Mood State

**Table 3:** Comparison of HIV+ and HIV- participants’ performance on the UPSA-B.

UPSA-B	HIV+ participants (n = 103), mean(SD); range	HIV- participants (n = 91), mean(SD)	*t*	*df*	*p*
Finance Subscale Score^1^	42.6(6.5); 23–50	44.3(5.0); 23–50	2.00	192	0.05
Communication Subscale Score^1^	37.0(8.5); 11–50	39.0(7.5); 22–50	1.80	192	0.07
UPSA-B Total Score^2^	79.6(11.4); 49–100	83.3(10.1); 51–100	2.40	192	0.02

Note. UPSA-B = UCSD Performance-Based Skills Assessment-Brief;

^1^Possible range of scores for each subscale: 0–50;

^2^Possible range for total score: 0–100.

### Associations between performance on the UPSA-B, neuropsychological performance, and self-reported IADL independence in HIV+ adults

Among the HIV+ participants, 40 (39%) met criteria for neuropsychological impairment (via GDS Impairment), and 27 participants (27%) met criteria for self-reported IADL dependence; 11 participants (28%) had both GDS impairment and IADL dependence. Among HIV+ participants, higher UPSA-B scores were related to higher education levels (r = 0.26, *p* = 0.01) and White race (r = 0.31, *p*<0.01), but were unrelated to age (r = 0.01, *p* = 0.96), gender (r = 0.03, *p* = 0.78), duration of HIV (r = 0.04, *p* = 0.66), CD4 counts (nadir CD4: r = 0.06, *p* = 0.54; current CD4: r = 0.09, *p* = 0.39), detectable viral loads (r = 0.06, *p* = 0.58), AIDS status (r = 0.07, *p* = 0.50), ARV status (r = 0.09, *p* = 0.40) or HCV serostatus (r = 0.05, *p* = 0.64). Lower UPSA-B total scores were related to lower premorbid intellectual functioning (i.e., WRAT Word Reading score; r = 0.27, *p*<0.01) and lower global neuropsychological scores (r = 0.41, *p*<0.001); in addition, lower UPSA-B scores were identified among those with GDS-derived neuropsychological impairment (M UPSA-B = 74.5, SD = 10.9) as compared to those without NP impairment (M UPSA-B = 82.8, SD = 10.6; *t* = 3.84, *p* < 0.001). UPSA-B total scores were unrelated to self-reported IADL independence vs. dependence (*t* = 0.93, *p* = 0.35) or POMS Confusion/Bewilderment scores (r = 0.17, *p* = 0.09).

Follow-up analyses examining the specific neurocognitive domains related to UPSA-B scores indicated positive, significant correlations at *p* < 0.05 between performance in all neurocognitive domains (verbal ability: r = 0.40, *p*<0.001; executive functions: r = 0.34, *p*<0.001; speed of information processing: r = 0.30, *p*<0.01; learning: r = 0.25, *p* = 0.01; recall: r = 0.21, *p* = 0.03; working memory: r = 0.45, *p*<0.01; motor ability: r = 0.22, *p* = 0.03).

### UPSA-B cut-point for predicting neuropsychological impairment in HIV+ adults

**Fig 1:**
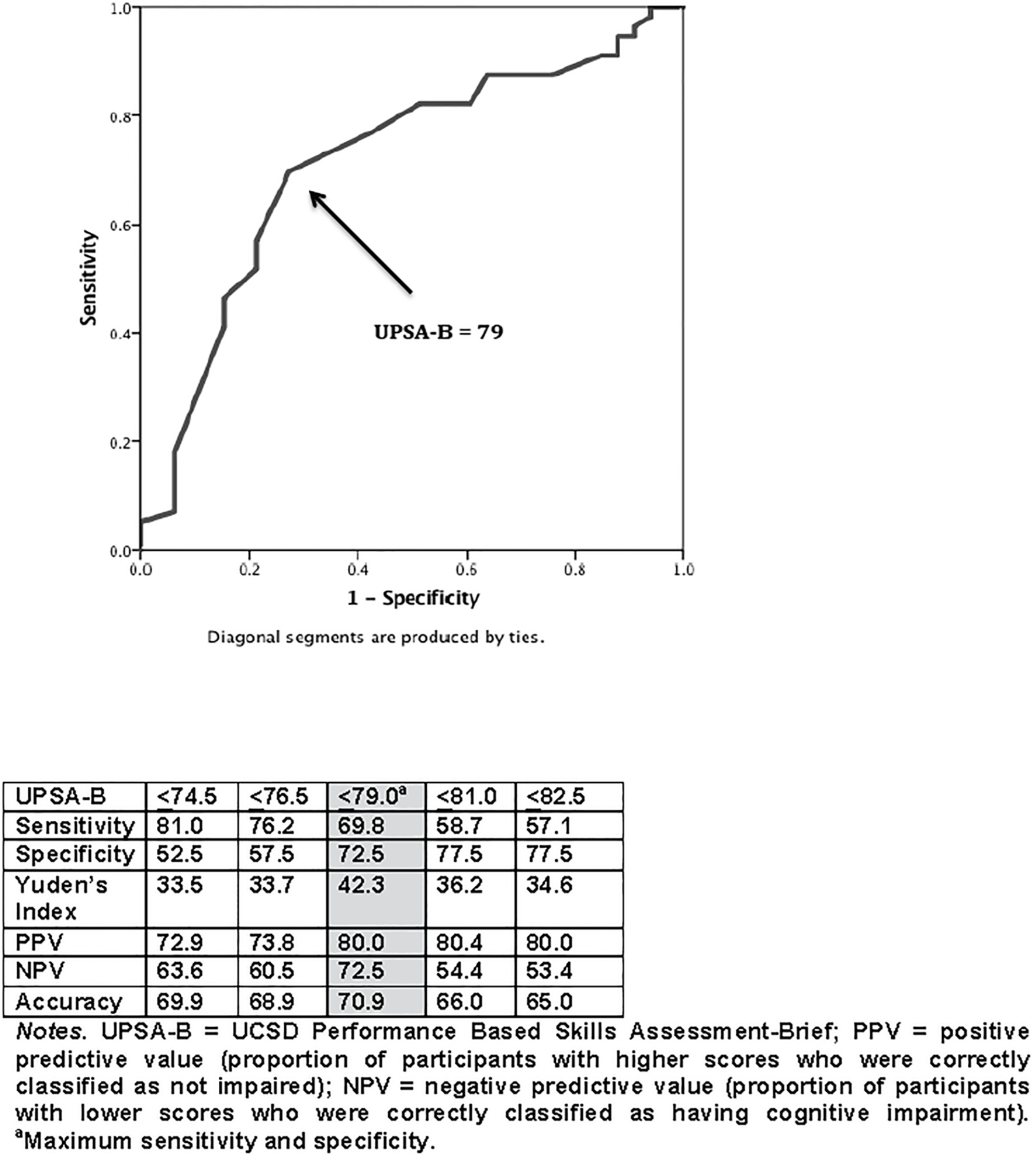
Receiver operating curve for UPSA-B predicting neurocognitive impairment.

In order to identify whether UPSA-B score can be an adequate predictor of neurocognitive impairment among HIV+ adults, we plotted a ROC curve for the UPSA-B to determine the optimal cut-point in discriminating between neurocognitive impairment vs. no impairment. The estimated area under the curve (AUC) for the UPSA-B was 0.72 (95% CI: 0.62–0.82), which was significantly greater (*p*<0.001) than the area of no information (AUC of 0.50). ROC analysis for the UPSA-B revealed a cut-point of 79 as the most optimal balance of sensitivity (69.8%) and specificity (72.5%) (see Fig 1).

## Discussion

Significant differences in UPSA-B performance were found between HIV+ and HIV- participants, although these results became nonsignficant when we controlled for group differences in education and sex. This finding suggests the UPSA-B may be a sensitive assessment of functional capacity among HIV+ adults, but future studies are needed to further evaluate UPSA-B differences among those with and without HIV before more definitive conclusions can be drawn. The UPSA was initially developed to assess functional capacity among individuals with serious mental illness, who generally have more prominent and severe cognitive and functional deficits than persons living with HIV. Future corroboration of our hypothesis with significant results, however, may indicate that the UPSA-B might be sensitive to the milder neurocognitive and functional impairments of people living with HIV/AIDS. Overall, there was a broad range of UPSA-B scores among our HIV+ participants (M = 79.6, SD = 11.4, range = 49–100), and previous work has demonstrated the UPSA-B is not a good indicator of functional capacity among individuals without neuropsychological impairments [39]. More challenging performance-based measures of functional capacity, such as the Advanced Finances Test [1] or the Multitask Test [2] may be more appropriate if the goal is to either characterize functional capacity among those with intact cognition or differentiate persons living with HIV/AIDS from persons without HIV/AIDS; however, this needs to be explored in future studies.

The UPSA-B appears to have utility in this population as a brief screener for potential neurocognitive impairment. Consistent with studies in other populations [40], and supporting the convergent validity of the UPSA-B with neurocognitive ability, we found global neuropsychological performance was related to functional capacity as measured by the UPSA-B. Examination of specific neuropsychological domains showed that the UPSA-B was associated with performance in all domains. Better UPSA-B performance was associated with higher education levels and White race, but was unrelated to age, gender, HIV disease characteristics, or HCV serostatus, suggesting that other instruments may be more appropriate in more diverse and/or lower educated cohorts.

While the UPSA-B has historically been used as an outcome measure for neurocognitive impairment or functional dependence, we chose to examine the use of the UPSA-B as a screening tool for neurocognitive impairment for clinical reasons. Adults living with HIV who are experiencing neurocognitive problems are more likely to seek treatment from their primary care physician or an infectious disease specialist than from a neuropsychologist. By design, the UPSA does not require a higher level degree, such as a Master’s or Doctorate degree, to administer and interpret (as opposed to neuropsychological tests), and could therefore be administered by staff at a primary care, infectious disease, or occupational therapy clinic. We found that the UPSA-B was able to differentiate HIV+ participants with and without neurocognitive impairment 70.9% of the time. The UPSA-B appears to be a sensitive neurocognitive screener, especially among those without cognitive impairment (positive predictive value [PPV] = 80.0%), and it has adequate ability to quickly identify persons living with HIV/AIDS who have possible cognitive impairment (negative predictive value [NPV] of 72.5%). Therefore, we recommend that patients with UPSA-B scores of ≤79 be referred for further neuropsychological evaluation to more accurately assess and determine presence of HAND.

### Limitations

Our study has several limitations. First, the data were cross-sectional, which limits our ability to make inferences regarding causality. Future work is needed to determine whether the UPSA-B could predict declines in cognition. Second, one quarter (25%) of the HIV- comparison participants met criteria for cognitive impairment, which is higher than the average population of HIV- adults. The substantial number of HIV- participants with cognitive impairment may have impacted the results, in that these were not a representative sample of comparison participants. In addition, our sample was relatively healthy from an HIV standpoint, with 94% of the HIV+ participants currently virally suppressed, which also limits the generalizability of our findings to less healthy HIV+ samples. The UPSA-B did not perform well for non-white and lower educated individuals, and there is a need for more ubiquitous but sensitive tasks of everyday functioning. Another limitation of our study is our IADL measure is a retrospective, self-report measure and categorizes people into IADL dependent or independent. Daily functioning impairments are often more subtle and can change over time in regard to completing complex daily activities, making these impairments difficult to detect. Additionally, our IADL self-report measure is limited in that the definition of “daily functioning” does not capture important domains of daily functioning beyond ADLs and IADLs, such as engagement in productive or cognitively stimulating activities, physical activities, or social activities. While performance-based tests of daily functioning, such as the UPSA-B, may provide a useful snapshot of functional capacity at one time point, it may not fully capture the intricacies or functioning demands of real-world daily functioning. Ambulatory data collection techniques that rely on repeated intra-day assessments of functional behaviors in real-time and in ecologically valid contexts, such as ecological momentary assessment (EMA), hold promise as a mobile assessment tool to assess daily functioning behaviors in HIV [41], and may be more appropriate than self-report measures or performance-based tests in certain contexts.

## Conclusions

Overall, functional disability among HIV+ adults is still not well understood and is likely multifactorial in nature. These data highlight the complex interplay between objective functional capacity, neurocognitive ability, subjective cognitive symptoms, and functional dependence. The UPSA-B statistically distinguished people with HIV/AIDS from comparison participants when education and sex were not taken into account; however, this finding was only at a trend level (*p* = 0.08) when these demographic differences were taken into account. On the other hand, and arguably more importantly, the UPSA-B was able to differentiate neurocognitive impairment from no impairment among the HIV+ participants. Differentially, our measure of self-reported cognitive difficulties was unrelated to UPSA-B performance, although there is a need for more sensitive self-report measures of cognitive difficulties [14,19,42]. These results emphasize the clinical importance of considering both objective and self-report data in the determination of a patient’s cognitive and functional abilities.
